# Reduced statherin in acquired enamel pellicle on eroded teeth compared to healthy teeth in the same subjects: An in-vivo study

**DOI:** 10.1371/journal.pone.0183660

**Published:** 2017-08-24

**Authors:** Mahdi Mutahar, Saoirse O’Toole, Guy Carpenter, David Bartlett, Manoharan Andiappan, Rebecca Moazzez

**Affiliations:** 1 Mucosal and Salivary Biology, King's College London Dental Institute, London, United Kingdom; 2 Tissue engineering and Biophotonics, King's College London Dental Institute, London, United Kingdom; 3 Biostatistics and Research Methods Centre, King's College London Dental Institute, London, United Kingdom; Virginia Commonwealth University, UNITED STATES

## Abstract

The aim of this in-vivo study was to compare total protein and four key salivary proteins present in the acquired enamel pellicle (AEP) on eroded and non-eroded surfaces in participants with erosive tooth wear. Participants with erosive tooth wear of dietary non-intrinsic origin, present on the occlusal surfaces of the lower first molars and an unaffected posterior occlusal surface in the same quadrant were recruited from restorative dental clinics at King’s College London Dental Institute (n = 29, REC ref 14/EM/1171). Following removal of the salivary film, AEP samples were collected from the eroded occlusal surfaces (EP, n = 29) and the non-eroded occlusal surfaces (NP, n = 29) using 0.5% sodium dodecyl sulfate (SDS) soaked filter papers. Total protein concentration was analysed using bicinchoninic acid assay (BCA). Protein fractions were separated using SDS-PAGE and immunoblotted against: mucin5b, albumin, carbonic anhydrase VI (CA VI) and statherin antibodies. Amounts were quantified using ImageLab software against purified protein standards of known concentration. ANOVA followed by paired t-test and Wilcoxon’s matched-pair signed-rank test were used to test statistical significance. The difference was considered to be significant at a P value < 0.05. The total protein on eroded surfaces was significantly lower compared to the total protein on non-eroded surfaces [0.41mg/mL (0.04) and 0.61 mg/mL (0.11)] respectively (p< 0.05). The median (min, max) amount of statherin was also significantly lower on eroded occlusal surfaces [84.1 (20.0, 221.8) ng] compared to AEP from non-eroded teeth in the same subjects [97.1(30.0, 755.6) ng] (p = 0.002). No statistical differences were observed for mucin 5b, albumin or CA VI. The total protein and statherin in the in-vivo AEP were different between eroded and non-eroded tooth surfaces of the same patient.

## Introduction

Erosive tooth wear has become a prevalent oral health problem affecting an increasing number of the population worldwide [1, 2]. It can lead to many clinical concerns such as poor appearance, loss of function, pain and discomfort [3]. Erosive tooth wear progression is influenced by a number of chemical, biological and behavioural factors [4, 5]. The protective role of salivary proteins and acquired enamel pellicle (AEP) against erosive tooth wear have been well documented [6–8]. AEP is a thin film of salivary proteins, which forms on oral soft and hard tissues by selective adsorption from whole mouth saliva. The role of AEP in preventing erosive tooth wear is hypothesised as a diffusion barrier that reduces direct contact between acids and the tooth surface [9–11], a neutralizer of protons [4, 12, 13] and a mineral reservoir that can potentially remineralise the demineralised tooth tissue [9, 14]. The protein components of in-vitro AEP have been studied by several authors [9, 15–20] including mucins (mucin5b and mucin7), amylase, albumin, carbonic anhydrase (CA IV), proline-rich proteins (PRPs), histatins, statherin, cystatins, SIgA, lysozyme and lactoferrin amongst others. Some of these proteins such as statherin and proline-rich proteins have been observed to adhere quickly and strongly with the enamel crystals [21, 22]. These proteins possess phosphate groups, which attract calcium and phosphate ions to the enamel surface. This potentially plays a role in the regulation of calcium phosphate homeostasis [23]. Other proteins such as mucins, amylase, albumin and CA VI build up later by the constant flow of saliva over tooth surfaces [24, 25] and serve many functions such as lubrication (mucins [25]) and diffusion barrier (albumin [26]) and acid neutralization (CA VI [17, 19]). Much of the work on protein composition has been carried out on in-vitro AEP which has provided some insight into their role in erosive tooth wear. However, there are many differences between in-vitro and in-vivo AEP due to the unique features of the oral environment such as the dynamics of salivary flow, enzymatic activities, thickness of the AEP, mineral surface properties and health and age of patients [18, 27]. In addition, in-vitro studies use ground and polished enamel surfaces which differ in susceptibility to acid challenge and enamel mineral content compared to the outer natural enamel layer [28, 29]. As stated above, calcium and phosphate ions of enamel crystals interact with the charged molecules of some salivary proteins so that the enamel surface layer is influenced by the type of proteins adsorbed into the AEP [27]. A limited number of studies have been carried out investigating the compositional differences of in-vivo AEP. These studies investigated the protein composition of AEP formed on permanent teeth [17,30–33] as well as on deciduous teeth [22, 32]. These studies were limited by the large variations between subjects within each group making comparisons difficult. Examples of these variations include the inter-subject variability in saliva and tooth structure. Most importantly, regional differences in salivary proteins caused by local topography and tribology may lead to inaccurate in-vivo results.

Previous work by this research group [33] compared mucin5b and CA VI in AEP between subjects with erosive tooth wear (n = 30) and healthy, age-matched controls (n = 30). Although reduced levels of total protein, statherin and calcium were observed in the AEP of participants with erosive tooth wear compared to healthy controls, no differences in other proteins (Mucin5b and CA VI) were detected. This is possibly attributed to the variability in saliva source, the starting protein concentration and rate of saliva replenishment between subjects. Thus the aim of this study was to investigate the protein composition of in-vivo AEP between eroded and non-eroded surfaces in the same participants. Due to the very small amounts of protein available, a targeted approach was used to measure four key salivary proteins: mucin5b, albumin, CA VI and statherin on an eroded tooth compared to a healthy tooth within the same subject.

## Material and methods

### Human subjects

Ethical approval was obtained from the national research ethics committee (East Midlands ethics committee, REC ref: 14/EM/1171). As previous studies for comparing mean protein levels [33, 34] showed a large effect size of 0.8, the power calculation for comparing the mean protein levels was carried out based on paired t test with an effect size of 0.6 and 80% power which yielded a total sample of 24 to test the difference at 5% level using two tailed test. Therefore, a total sample of 29 were recruited. The mean age for participants was 37.7 years (SD = 11.7, Range 25–61). More females were recruited [17 (58.6%)] than males [12 (41.4%)]. The baseline mean total BEWE score was 14.7 (SD = 2.5). The source population were participants of both sexes referred in via their general dental practitioner for treatment at the restorative clinics in King’s College London Dental Institute, Guy’s hospital London. Patients presenting with moderate to severe erosive tooth wear were invited to take part in a screening examination. Following informed written consent, dental and medical histories were checked and a Basic Erosive Wear Examination (BEWE) was used to assess erosive tooth wear [35]. Participants were included in the study based on the following criteria: age 25–61 years, a minimum of 20 teeth (10 in each jaw), a BEWE score of 3 on the occlusal surface of the lower molars or the incisal edge of the upper central incisors and a cumulative BEWE score ≥ 8. Wear had to be a result of a high acid diet (≥ daily dietary acid challenges). Participants were excluded from the study based on the following criteria: medical or dental problems such as severe dentine hypersensitivity, periodontitis or restoration of the occlusal or incisal surfaces of upper anterior teeth and first molars, missing anterior teeth, anterior crowns/bridges or cavitated caries on more than one tooth, history of eating disorders, gastro-oesophageal reflux, xerostomia, bruxism, prescribed xerostomic/heartburn medication, pregnancy, participation in other research studies in the last 30 days, inability to speak or understand the English language and necessity of taking antibiotics prior to dental visits/procedures.

### Sample collection

A single trained and calibrated investigator performed all wear and dietary assessments in addition to AEP collection with the patient in a reclined position and good lighting. Participants refrained from eating and drinking at least 1 h prior to AEP collection. Selected teeth were isolated with cotton wool rolls and filter paper was applied to occlusal surfaces to clear the salivary film. The collection of AEP was standardised through the use of a standardised size filter paper (21 mm X 3 mm) and standardised method of collection by a single investigator. Using sterile tweezers to hold a filter paper soaked in sodium dodecyl sulfate (SDS) (0.5% w/v), AEP was collected by mechanically agitating the paper uniformly against a localised section of the surface (3 x 3 mm) for a timed period of 15 seconds as per previous protocols [9, 31, 33, 36].

A total of one hundred and sixteen AEP samples were collected from twenty nine erosion patients. 58 AEP samples from eroded occlusal surface of the lower first molars (n = 58, 100%) and 58 AEP samples from non-eroded adjacent posterior occlusal surface [premolars (n = 21, 36%) and molars (n = 37, 64%)]. AEP samples were collected from four surfaces in each patient, one eroded and one non-eroded adjacent posterior occlusal surface in each of the lower left and right sextants. The two eroded samples obtained from the same patient were pooled to be analysed together producing a total of 29 eroded AEP samples (EP). The two non-eroded AEP samples from each patient were also pooled to be analysed together producing a total of 29 non-eroded AEP samples (NP). Analysis was performed by an investigator blinded to the erosion status of the sample surface.

### In-vivo AEP harvest and recovery

Filter papers carrying the AEP were microcentrifuged and the adsorbed proteins were recovered by adding 15 μl SDS (Sigma-Aldrich, Steinheim, Germany) (0.5%) and 5 μl LDS buffer (1:4) (Novex, Thermo Fisher Scientific Inc, UK). The AEP eluent were microcentrifuged for 8 min at 8000 rpm and dithiothreitol (DTT) (1.8 μl, 0.5 mM) reducing agent (1:10) (Sigma-Aldrich, Poole, Dorset, UK) was added to the eluent. Samples were vortexed for 1 min with a vortex mixer and then heat denatured at 100°C for 5 min.

### Testing

#### Total protein in AEP

The AEP samples were prepared into microtiter plates (96-wells, Fisher Scientific, Leicestershire) and the total protein was measured using the bicinchoninic acid assay (BCA) with bovine serum albumin (BSA) protein as a standard protein (2 mg/mL) (Pierce Chemical, Rockford, Ill., USA). A spectrophotometer (BioRad laboratories Ltd, Hemel Hempstead, UK) at wavelength of 562 nm was used to measure the absorbance of all samples.

#### Electrophoresis

**Fig 1:**
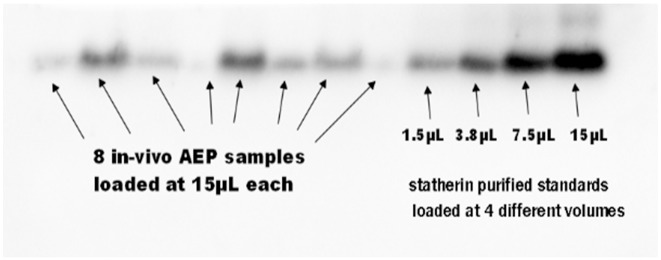
An example of SDS-PAGE and western blotting of AEP samples loaded at 15 μL and purified standards loaded at 4 different volumes.

Sodium dodecyl sulphate–polyacrylamide gel electrophoresis (SDS–PAGE) was used for separation of protein fractions in their denatured state from the recovered AEP samples. Each AEP prepared sample was equally loaded (15 μL) onto each lane on a 4–12% Bis-Tris gel. Electrophoresis was carried out in MES-SDS running buffer according to manufacturer’s instructions. In each gel, 8 lanes were occupied by the AEP samples and the other 4 lanes were occupied by a mixture of four purified proteins of standards of known concentration. The volumes of the purified standards were 15 μL/lane 1, 7.5 μL/lane 2, 3.8 μL/lane 3 and 1.5 μL/lane 4 as shown in Fig 1.

The purified standards used in the mixture were mucin5b (156 μg/ml) (Kind gift of Faculty of Odontology, Malmö University, Malmö, Sweden), albumin (1 μg/ml) (Alpha Diagnostic IntL. Inc, San Antonio, Texas 78244 USA), CA VI (140 μg /ml) (Jena Bioscience, D-07749 Jena Germany) and statherin (382 μg/ml). Statherin was prepared by the author as described previously by Proctor et al, (2005) and Harvey et al, (2011) [14, 37]. Fresh human parotid saliva from a single donor at King’s College London was collected and was aliquoted into 10 petri-dishes, 1 ml each and exposed to air to form a statherin-rich film at the air/saliva interface after 1 h. The residual saliva underneath the film was carefully pipetted out and washed three times with 100 μl of deionised water. A 100 μL wash of 10 mM EDTA was then added to solubilise the statherin layer which was then separated and tested for identity and purity using SDS-PAGE gel stained with Coomassie Brilliant Blue and antibody detection [14, 37]. The statherin content in the purified film protein was 382 μg/ml.

The volume of purified proteins used in the mixture were mucin5b (10 μL), albumin (10 μL), CA VI (5 μL) and statherin (5 μL) to make a 30 μL mixture of purified standards optimised to give a linear standard curve with the antibodies used. This was duplicated ten times to produce a 300 μl mixture to generate sufficient amounts for the 15 SDS-PAGE gels. The loaded protein samples in the precast gels were then transferred onto a nitrocellulose membrane using western blot technique.

#### Immunoblotting

Western blotting was completed according to the manufacturer’s instructions and used to transfer proteins onto a nitrocellulose membrane. Using a sterile razor, each nitrocellulose membrane was cut transversely into four sections corresponding to the four specific proteins of interest. At room temperature, the nitrocellulose membranes were then blocked in Tris Tween buffer solution with 1% Tween (TTBS) pH 7.6 for 1 h before membranes were probed with primary antibodies: mucin5b (1:1000) (GENTAUR Ltd. 1910 kampenhout, Belgium), albumin (1:1000) Sigma-Aldrich, Saint Louis, MO 63103 USA), carbonic anhydrase VI (CA VI) (1:5000) (R&D Systems UK Abingdon, OX14 3NB, UK) and statherin (1:1000) (a gift from Paul Anderson, QMUL, London, UK). The nitrocellulose membranes were then washed in TTBS for 15 min (5 min X 3 times) and then followed by incubation with the required secondary antibody. A final 15-min wash in TTBS was completed before the membranes were imaged.

#### Imaging analysis of the blotted membrane

ChemiDoc MP imaging analysis (Bio-Rad) was used to quantify the light intensity of the chemiluminescent reaction and exposure times optimised to prevent pixel saturation. The amounts of proteins on the blotted nitrocellulose membranes were quantified using tools of ImageLab software version 4.1 (Bio-Rad Laboratories Ltd., Hertfordshire, UK) to select and determine the background-subtracted density of the bands in all the gels (n = 15) using purified protein standards of known concentration. The standard curves of purified proteins were generated from the mean volume intensities (n = 15) against the absolute quantities of the corresponding purified standard. This was used to generate a calibration curve (S1 Fig) using a linear formula. Curves were generated from volume intensities (mean±SD) against the absolute quantity in nanogram (n = 15) and were used to quantify the absolute quantity of proteins in the AEP samples. This formula was used to calculate the amount of each protein in the AEP samples. The bands of standard proteins on different SDS-PAGE gels (n = 15) were used to assess reproducibility.

### Statistical analysis

Statistical analysis was performed using IBM SPSS Statistics version 23.0 (IBM Corporation, Armonk, New York). Data were assessed for normality using histograms, boxplots and Shapiro-Wilks tests. Data for total protein, albumin and CA VI were observed to be normally distributed, therefore data were analysed using paired t-test and presented as mean and standard deviation (SD). Data for mucin5b and statherin did not follow normal distribution, therefore data were analysed using Wilcoxon’s matched-pair signed-rank test and presented as median (min, max) and. The level of significance was set at a value of p < 0.05.

## Results

The total protein concentration (SD) on the eroded surfaces (n = 29) was [0.41 (0.04) mg/mL] and on the non-eroded surfaces (n = 29) [0.61 (0.11) mg/mL]. This difference was statistically significant (p< 0.05).

Table 1 reports the mean (SD) of albumin and CA VI as well as the median (min, max) amount of statherin and mucin5b, in nanograms. The median (min, max) amount of statherin collected on the eroded occlusal surfaces was [84.1 (20.0, 221.8) ng] and on non-eroded surfaces in the same subjects was [97.1 (30.0, 755.6) ng]. This difference was statistically significant (p = 0.002). The median amount of mucin5b (min, max) collected from eroded surfaces was [96.0 (80.0, 328.2) ng] and from non-eroded surfaces [96.0 (40.7, 574.5) ng]. This difference was not statistically significant. The mean amount of albumin (SD) collected from eroded teeth surfaces was [3.8 (1.9) ng] and from non-eroded surfaces was [3.7 (1.7) ng]. This difference was not statistically significant. The mean amount of CA VI (SD) collected from eroded teeth surfaces was [60.8 (49.6) ng] and from non-eroded surfaces was [101.3 (72.3) ng]. This again, was not statistically significant.

**Table 1:** Amount of four proteins (ng) in pellicle (P) samples.

Protein investigated	Eroded surfaces pellicle (EP)		Non-eroded surfaces pellicle (NP)		Sig
	Mean (SD)	Median (min, max)	Mean (SD)	Median (min, max)	
**Statherin (ng)**	90.1 (42.6) (n = 23)	84.1 (20.0, 221.8) (n = 23)	167.2 (186.3) (n = 27)	97.1(30.0, 755.6) (n = 27)	p = 0.002 a
**Mucin5b (ng)**	112.3 (65.2) (n = 13)	96.0 (80.0, 328.2) (n = 13)	119.9 (114.6) (n = 18)	96.0 (40.7, 574.5) (n = 18)	p = 0.878 a
**Albumin (ng)**	3.8 (1.9) (n = 29)	3.8 (0.9, 9.3) (n = 29)	3.7 (1.7) (n = 29)	3.7 (1.1, 6.7) (n = 29)	p = 0.702 b
**Carbonic anhydrase VI (ng)**	60.8 (49.6) (n = 27)	48.1 (0.2, 207.2) (n = 27)	101.3 (72.3) (n = 24)	69.5 (25.0, 262.5) (n = 24)	p = 0.059 b

AEP samples were eluted from eroded (E) and non-eroded (N) tooth surfaces using 0.5% SDS and quantified using ImageLab software using purified protein standards. P values were calculated using ^a^ paired t test or ^b^ Wilcoxon’s matched-pair signed-rank test.

## Discussion

The total AEP protein concentration was observed to be significantly lower on eroded occlusal surfaces compared to non-eroded surfaces in the same sextant and within the same patient. In addition, the amount of statherin was significantly lower in the AEP of eroded surfaces compared to statherin amounts observed in the AEP of non-eroded surfaces. Mucin5b, albumin and CA VI were detected in the AEP of eroded and non-eroded teeth surfaces of the same patient but the amounts did not differ significantly between the surfaces.

To the author’s knowledge, no in-vivo AEP studies have quantified mucin5b, albumin and CA VI. Only a few studies [19, 33] have directly quantified statherin. Interestingly, part of in-vivo AEP remains in place even after severe erosive challenges, indicating that some proteins remain in place and have the potential to prevent erosive tooth wear [22, 38]. In the present study, the selection of the four proteins was based on their hypothesized mechanisms of actions against erosive tooth wear. These included the physical permeable barrier and lubrication properties of mucin5b and albumin, buffering capacity of CA VI and calcium binding mechanism of statherin. In addition, albumin is also believed to bind to calcium ions in the enamel crystals [26] but such affinity for hydroxyapatite was reported to be low [9].

The results of our study agree with a previous study carried out by Carpenter et al, (2014) who compared the levels of total proteins and statherin between thirty participants with and without erosive tooth wear [33]. Both studies agree that total proteins concentration and amount of statherin in AEP were lower from patients with erosive tooth wear, or as is the case in the current study, from eroded surfaces than that from healthy subjects, or non-eroded surfaces. However, Carpenter et al.(2014) investigated the difference in the amount of mucin5b and CA VI in resting saliva but not in AEP from participants with and without erosive tooth wear [33]. They demonstrated that saliva from erosion patients had reduced amounts of mucin5b and CA VI compared to patients without erosive tooth wear. This is different from results observed in our study on the AEP whereby the amounts of mucin5b, CA VI and albumin in AEP were not significantly different between eroded and non-eroded surfaces in the current study. This can be explained by the variability between subjects as Carpenter et al., (2014) compared these proteins in saliva between healthy subjects and subjects with erosive wear whereas our current study compared the proteins in AEP of the same subject. Saliva source, composition and rate of saliva replenishment differ between subjects but not within the same subject. Based on the results from this study, there is not a clear cut explanation to this but it seems that the differences in mucin5b and CA VI found in saliva but not in AEP between healthy subjects and subjects with erosive wear is more likely attributed to the difference in absorption, degradation, clearance of those particular proteins, since WMS is the residual saliva after the secreted glandular saliva is adsorbed into the hard and soft oral tissues. Absorption of proteins to surfaces is a highly variable process, dependent on many factors such as the adsorbent surface area, surface charge, counter ions and pH, some of which our research group continues to investigate. Our study also suggests that specifically statherin is not adsorbing to the eroded tooth surface as well as the other proteins studied. Combining our results with that of Carpenter et al, (2014), the level of mucin5b and CA VI may suggest that the delivery of these two proteins from salivary film to the AEP as well as the flow and viscosity properties of the salivary film are important to the quality of AEP [33]. Albumin is abundant in AEP and amongst the first proteins to adsorb to enamel [31]. Siqueira et al, (2012) suggested that albumin may be less likely to alter structurally or functionally in the mouth before they are incorporated into the AEP [31]. This agrees with the results in this study that albumin has the same affinity to eroded and non-eroded surfaces and that neither the saliva status nor the surface topography altered its adsorption.

The inherent protection against erosive tooth wear may be dependent on individual AEP proteins, in combination with the properties of enamel substrate including topography, tribology and surface roughness. This study may suggest that saliva may deliver proteins e.g statherin more effectively to non-eroded compared to eroded surfaces. Statherin was found to be amongst the first AEP proteins to be adhered competitively to hydroxyapatite [31] which supports the idea of favourable adsorption of statherin onto such tooth surfaces compared to other salivary proteins. The increased level of statherin, a calcium binding protein, on non-eroded surfaces indicates that calcium and phosphorus ions are possibly modulated around the enamel crystals and that statherin is potentially a major mediator against erosive tooth wear and that AEP protection in erosive tooth wear is over-ridden by altered calcium homeostasis rather than a failure to neutralise or block acidic attack. Another possible explanation is that the adsorbed layer of statherin on the non-eroded surfaces may modify the adhesive and lubrication properties of the non-eroded surfaces [37]. This could change its tribology influencing the wear and friction properties of the surface. Saliva/enamel interaction and AEP formation are influenced by the surface roughness, surface free energy, surface chemical composition, wettability and many other interaction forces [39, 36]. In this regard, the competitive absorption of statherin onto non-eroded surfaces may also have influenced the adsorption of other proteins due to the competition and synergism among all in-vivo AEP proteins during the dynamic event of AEP formation. Statherin with histatins and PRPs act as the AEP precursors whereby they are cross-linked by the enzymes altering the surface of the formed pellicle into hydrophobic which act as a template for further protein assembly while preventing other proteins from binding [40]. Although the data in the literature on surface texture is still contradictory, it is generally understood that erosive challenges increase enamel roughness to a certain degree before smoothing of the surface takes place [41]. With regard to roughness, the greater intake of acidic food and drinks in erosion patients is likely to cause clinical signs of erosive tooth wear such as roughened or smooth surfaces which may change the surface binding affinity to certain proteins, including statherin. Further investigation with longitudinal studies is necessary to establish the interaction between tribology and topography of the tooth surface.

The salivary film was initially removed from the underlying AEP in order to assess the amount of the various proteins more accurately. A well-established method of collecting AEP from tooth surfaces was also applied in order to remove all organic materials from the enamel surfaces and avoid any contamination [20, 33, 42]. Looking at the median (min, max) amount of statherin reported in the present study [84.1 (20.0, 221.8], it can be seen that it is comparable to that reported in previously published results [36.2 (17.0, 75.9) ng], suggesting that the sampling method is at least as consistent as previously published study [33]. The current study has some limitations. Calcium ions in AEP samples were not analysed in this study due to the small amount of fluid collected. In addition, proteomic analysis of AEP at a larger scale was not feasible due to cost and time limitations. The relatively small sample size of 29 patients recruited for this study was however compensated for by the experimental design where comparisons were made between several samples obtained from the same participant. This reduces inter-subject variation that could cause bias such as diet and lifestyle, age, reflux, salivary properties, local topography of tooth surfaces. Participants with intrinsic source of acid such gastro-oesophageal reflux disease (GORD) were also excluded as their protein profile may be very different. The lower total protein concentration and statherin levels observed in the AEP were collected solely from eroded occlusal surfaces of the lower first molars and compared to non-eroded surfaces in the same posterior sextant. However, this was not verified with other eroded surfaces which is a limitation of the study. Future work could compare protein concentrations on other eroded versus non-eroded surfaces in different sextants but within the same patient to attempt to replicate these findings.

There is potential that a reduced amount of statherin on a tooth surface may serve as a biomarker for risk of erosive tooth wear progression, although further studies with a larger number of participants are needed to confirm these preliminary results. Other similar studies are required to investigate other salivary proteins which may also play the role in the protection against erosive tooth wear, potentially improving oral diagnostic, therapeutic and preventive measures. Further research could also focus on the adsorption behaviour of other individual proteins within AEP to different enamel surfaces.

## Conclusion

Total protein concentration in the AEP was reduced on eroded dental surfaces compared to non-eroded dental surfaces in the same subjects. Reduced amounts of calcium-binding statherin were also observed on eroded tooth surfaces. Results from this study imply that the protective role of saliva in erosive tooth wear is predominantly through calcium homeostasis rather than a failure to neutralise or block acidic attack. Absence of statherin in the AEP may be a potential predictor for future erosive tooth wear. However, further investigation, both in-vitro and in-vivo are necessary to confirm these results.

## Supporting information

S1 Fig Standard curves of the purified proteins of standards. (A) Curves were generated from volume intensities (mean±SD) against the absolute quantity in nanogram (n = 15) and were used to quantify the absolute quantity of proteins in the AEP samples.
(TIF)
