# Supplementary figures and images for: Reduced statherin in acquired enamel pellicle on eroded teeth compared to healthy teeth in the same subjects: An in-vivo study
Source: PLoS One. 2017 Aug 24;12(8):e0183660. doi: 10.1371/journal.pone.0183660 (PMC5570300; doi:10.1371/journal.pone.0183660)

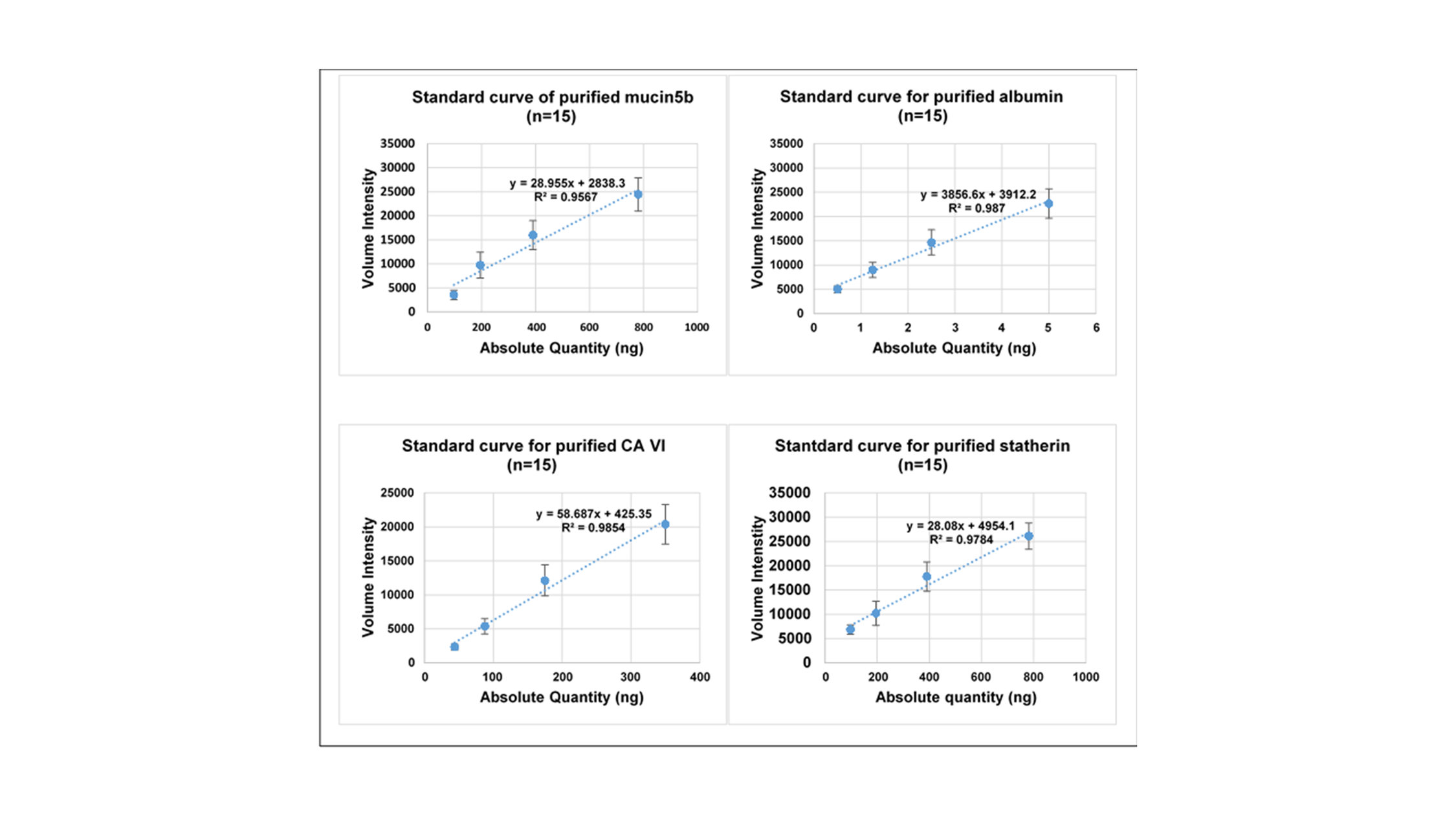

Supplement: S1 Fig — (A) Curves were generated from volume intensities (mean±SD) against the absolute quantity in nanogram (n = 15) and were used to quantify the absolute quantity of proteins in the AEP samples. (TIF) [file pone.0183660.s001.tif]
